# Elavl1 deletion in limb mesenchyme is dispensable for skeletal morphogenesis

**DOI:** 10.3389/fcell.2025.1501837

**Published:** 2025-07-14

**Authors:** Rohini Parsha, Satya K. Kota

**Affiliations:** Department of Oral Medicine, Infection and Immunity, Harvard School of Dental Medicine, Harvard University, Boston, MA, United States

**Keywords:** limb development, RNA-binding proteins, embryonic lethal abnormal vision-like protein 1/human antigen R, appendicular skeletal elements, RNA sequencing, Prx1-cre, Embryonic development

## Abstract

Embryonic lethal abnormal vision-like protein 1 (Elavl1)/human antigen R (HuR) is an RNA-binding protein implicated in multiple developmental processes, with pleiotropic roles in the RNA life cycle. Early embryonic loss of Elavl1 in epiblast cells is lethal due to defects in placental branching and embryonic tissue growth. Postnatal global deletion of Elavl1/HuR results in lethality with atrophy in multiple tissues, mainly due to the loss of progenitor cells. However, the roles of Elavl1 specifically during embryonic limb skeletal development are not well understood. In this study, we report that the deletion of Elavl1 in limb bud mesenchyme in mice did not reveal any abnormalities during embryonic development, with normal development observed in pre- and postnatal limb skeletons. Analyses of skeletal patterning, morphogenesis, and skeletal maturation, including skeletal elements in the stylopod, zeugopod, and autopod, during development did not reveal any significant differences between long bones from control and Elavl1 conditional knockout (cKO) animals. Our study indicates differential dependency and susceptibility to the loss of Elavl1 in different stem cell lineages, with its functions being dispensable during limb skeletal development.

## Introduction

RNA-binding proteins play very important roles in maintaining organismal homeostasis and mediating responses during disease resolution (Hentze et al., 2018). Embryonic lethal abnormal vision-like protein 1 (Elavl1)/human antigen R (HuR) protein plays important roles in controlling the cellular levels and stability of multiple AU- or U-rich RNA transcripts (Simone and Keene, 2013). Elavl1 regulation of RNA stability generally occurs via interactions with AU-rich elements (AREs) in the introns and 3′ untranslated regions (UTRs) of messenger RNAs (Lebedeva et al., 2011; Mukherjee et al., 2011). Interactions between ARE-containing mRNAs in the nucleus and Elavl1, in general, lead to stability by preventing access of these mRNAs to RNA-binding proteins that recruit additional proteins involved in the RNA decay pathway (Fan and Steitz, 1998; Brennan and Steitz, 2001; Gallouzi et al., 2001). In humans and other mammals, the Elavl family comprises four members, namely, ubiquitously expressed HuR/Elavl1 and developmentally regulated HuB/Elavl2, HuC/Elavl3, and HuD/Elavl4 (King et al., 1994; Good, 1995; Ma et al., 1996). All Elavl family members contain RNA recognition motifs (RRMs) that bind to U- and AU-rich sequences in RNAs, predominantly in the UTR regions (Millard et al., 2000; Peng et al., 1998). Ubiquitous distribution of Elavl1 points to fundamental roles in posttranscriptional regulation of RNA in multiple tissues during tissue homeostasis and development.

Elavl1 and its homologs regulate RNA metabolism and stability in many eukaryotic species, from *Drosophila* to humans (Cai et al., 2022). The deletion of HuR in *Drosophila* resulted in embryonic lethality with developmental defects, including in the nervous system (Campos et al., 1985). In mice, both pre- and postnatal deletion of Elavl1 leads to lethality. Deletion of Elavl1 using Sox2-cre led to defective development, with embryos showing skeletal and splenic defects and embryonic lethality (Kats et al., 2009). Postnatal global deletion of Elavl1 using tamoxifen-inducible Cre mice at 8 weeks of age also resulted in the atrophy of multiple tissues and lethality within 10 days of Elavl1 depletion. Furthermore, apoptosis of progenitor cells residing in multiple tissues, including bone marrow, thymus, and intestine, was detected upon the depletion of Elavl1 postnatally (Ghosh et al., 2009). Recently, Elavl1 was also found to be essential in developing cranial neural crest (Hutchins et al., 2022) and as a key protein for controlling hepatic metabolic homeostasis (Subramanian et al., 2022). *In vitro*, in cultured bone marrow stromal cells (BMSCs), Elavl1 adversely affected osteogenic differentiation by controlling stability and cellular expression levels of several ARE-containing mRNAs associated with ECM organization (Kota et al., 2021). In contrast to the above-known roles in multiple tissue-specific functions, very little is known about the role of Elavl1 during skeletal development. With the aim of understanding the role of Elavl1 during limb skeletal development, we generated Elavl1 conditional knockout (cKO) mice using Prx1-cre, which is expressed in the limb bud and a subset of cranial mesenchyme. Our results indicated that Elavl1 is expressed in developing limbs; however, loss of its expression in a specific and conditional manner in Prx1-cre-expressing cells was compatible with life and led to normal skeletal development, as analyzed across multiple stages during and after embryonic development.

## Methods

### Mouse crosses

All experiments involving mice were approved by the institutional IACUC. *Prx1*-cre [B6.Cg-Tg(Prrx1-cre)1Cjt/J, Strain #:005584] male mice, which carry a transgene with the Prrx1 promoter/enhancer sequence directing Cre recombinase expression in early limb bud mesenchyme and a subset of craniofacial mesenchyme, and Elavl1 floxed mice (Ghosh et al., 2009) (B6.129-Elavl1^tm1Thla^/J, Strain #:021431) with loxP sites flanking exons 2–5 were obtained from The Jackson Laboratory. Mice were housed in sterile cages with *ad libitum* access to food and water, under a 12-h light/12-h dark cycle. Male Prx1-cre (Bar Harbor, ME, USA) and female Elavl1 foxed mice were bred to generate Prx1-cre; Elavl1fl/fl embryos and mice. Elavl1fl/fl and Prx1-cre; Elavl1fl/+ embryos and mice were used as controls. Genotyping was performed with DNA isolated from ear punch tissues using the following primers for Elavl1: forward-CTC TCC AGG CAG ATG AGC A and reverse-TAG GCT CTG GGA TGA AAC CT.

### Embryo collection and skeletal preparations

Time-mated pregnant mice were euthanized at the indicated timepoints to collect the embryos at different developmental stages. Alcian blue and Alizarin red staining of skeletal preparations was performed similarly to that described by Rigueur and Lyons (2014). Forelimb, hindlimb, and cranial skeletons were dissected from the whole-mount skeletal preparations and imaged separately. Analysis was performed to assess the presence and maturation status of individual bones in stylopod, autopod, and zeugopod of forelimb and hindlimb skeletal elements.

### Real-time qPCR

Total RNA was prepared from hindlimbs collected from 12.5-day post coitum (dpc) embryos using the RNeasy Kit (QIAGEN, MD, USA). DNA contamination was eliminated through DNase I digestion. Complementary DNA was prepared using the multiscript reverse transcription system (Applied Biosystems, CA, USA). The reverse-transcribed cDNA was subjected to qPCR using a SYBR green-based detection system (QIAGEN). Relative levels of transcripts were normalized to beta-actin levels and quantified based on the 2^−ΔΔCT^ method. A minimum of three hindlimbs from cKO mice (Prx1-cre; Elavl1fl/+) or controls (Elavl1fl/fl and Prx1-cre; Elavl1fl/+) were analyzed. The primer sequences used will be available upon request.

### Analysis of Elavl gene expression and open chromatin at regulatory regions

Transcripts per million (posterior_mean_count) values for Elavl1, Elavl2, Elavl3, and Elavl4 were extracted from the poly-A plus RNA sequencing data from ENCODE (Consortium, 2012; Luo et al., 2020) (ENCSR098WGB, ENCSR407MLM, and ENCSR902MLV) that were obtained from 10.5 dpc, 11.5 dpc, 12.5 dpc, 13.5 dpc, 14.5 dpc, and 15.5 dpc limb RNA pooled from embryonic forelimb and hindlimb tissues. DNase-seq data peaks pertaining to Elavl1 promoter and genic regions for 10.5 dpc, 11.5 dpc, and 14.5 dpc limbs were obtained from ENCODE (ENCSR466MZF).

### Limb, femur, and tibial length measurement

Lengths of left and right forelimbs and hindlimbs from control and Elavl1 conditional knockouts from four-week-old mice were measured along the stylopod, zeugopod, and autopod using a digital Vernier caliper. Tibias and femurs were collected from mice hindlimbs, and proximo-distal lengths were measured using a Vernier caliper.

## Results

**FIGURE 1:**
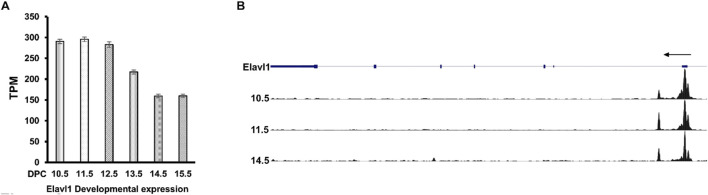
**(A)** RNA-seq analysis of messenger RNA levels of Elavl1 in limb RNA across developmental stages 10.5 dpc–15.5 dpc, indicating expression observed across these developmental stages. **(B)** DNase1-seq analysis of chromatin at 10.5 dpc, 11.5 dpc, and 14.5 dpc developmental stages shows DNase I hypersensitive peaks, indicating open chromatin near promoter regions of the *Elavl1* gene.

To understand the biological role of Elavl1 during limb skeletal development, Elavl1 gene expression was analyzed during mouse limb development between 10.5 dpc and 15.5 dpc from poly-A plus RNA sequencing data from ENCODE. Elavl1 mRNA expression was detected in all the developmental stages analyzed, with the highest expression observed at the 10.5 dpc limb bud stage. Compared to 10.5 limb buds, a gradual decrease in Elavl1 transcript levels was observed between embryonic stages 12.5 and 15.5 dpc (Figure 1A). The presence of Elavl1 mRNA levels correlated with open chromatin at the Elavl1 regulatory regions during the developmental stages (Figure 1B). In addition, single-cell RNA (scRNA)-sequencing data (ENCSR062VQC) from embryonic forelimbs showed that Elavl1 is broadly expressed in multiple cell lineages, including in mesenchymal, chondrocyte, and osteoblast lineages (Supplementary Figure 1).

**FIGURE 2:**
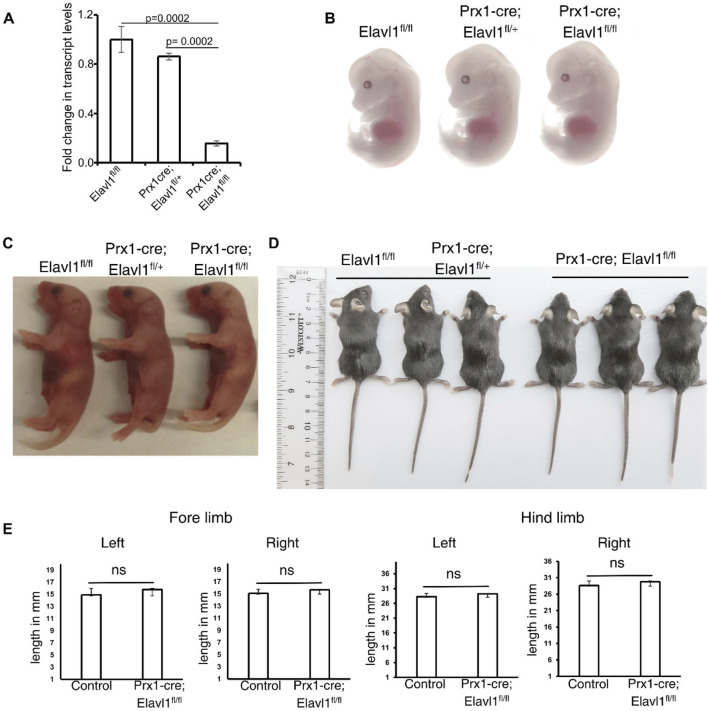
**(A)** RT-qPCR analysis of total RNA from control (N = 3) and Elavl1 conditional knockout limbs (N = 3) from 12.5 dpc embryos, indicating significant downregulation of Elavl1 RNA levels in limbs upon Prx1-cre-mediated deletion. Representative whole-body images of 13.5 dpc embryos **(B)** and neonatal mouse pups of P1 stages **(C)** of control (Elavl1^fl/fl^ and Prx1-cre; Elavl1^fl/+^, N = 8); Elavl1 conditional knockout (Prx1-cre; Elavl1^fl/fl^, N = 7) showing no gross morphological differences during pre- and postnatal development. Whole-body images of 4-week-old male control (Elavl1^fl/fl^ and Prx1-cre; Elavl1^fl/+^, N = 3) and Elavl1 cKO (N = 3) mice showing no gross morphological **(D)** differences including limbs. **(E)** Forelimb and hindlimb lengths in the proximal–distal axis in four-week-old male mice measured between control (n = 3) and Prx1-cre; Elavl1^fl/fl^ mice (n = 3), ns, not significant.

The expression data analysis confirmed the presence of Elavl1 in the developing limb bud. To investigate the role of Elavl1 during appendicular skeletal development, the exons 2–5 of the *Elavl1* gene were conditionally deleted from the early limb bud mesenchyme in Elavl1 floxed mice using Cre recombinase, driven by the Prx1 promoter/enhancer elements (Prx1-Cre) (Supplementary Figure 2). Both control and Prx-1-cre-mediated Elavl1 cKO mice were obtained in expected Mendelian ratios and survived to adulthood. RNA expression analysis from control and Elavl1 cKO embryonic hindlimbs using RT-qPCR indicated a significant reduction in Elavl1 mRNA levels in Elavl1 cKO embryonic limbs compared to littermate controls at 12.5 dpc (Figure 2A).

Next, to further understand the effects of Elavl1 depletion on appendicular skeletal morphology, morphological analyses of control and Elavl1 cKO 13.5 dpc embryos (Figure 2B), P1 pups (Figure 2C), and 4-week-old mice (Figure 2D) were performed. Gross morphological analysis did not reveal any significant differences between control and Elavl1 conditional knockout embryos or adult mice. Quantitation of forelimb and hindlimb lengths from four-week-old mice also did not reveal any significant differences between control and Elavl1 cKO mice (Figure 2E).

**FIGURE 3:**
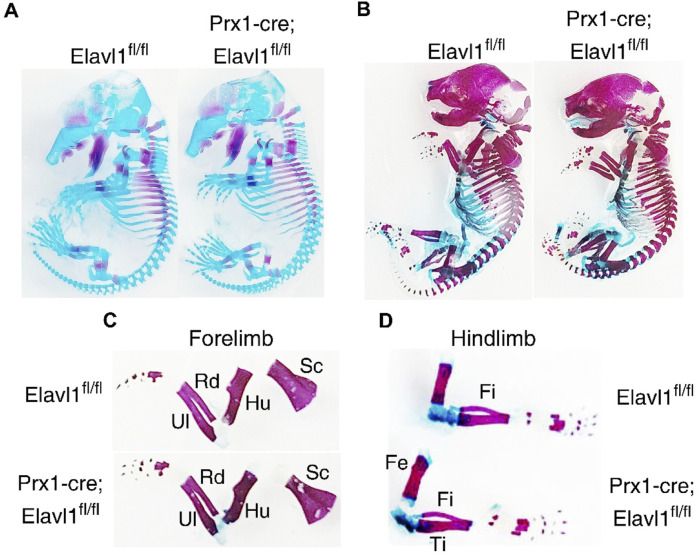
Representative whole-body skeletal preparations from 15.5 dpc **(A)** and 18.5 dpc **(B)** embryos, showing no apparent changes in stage-specific cartilage and bone staining between control and Elavl1 cKO mice at these embryonic stages. Forelimb **(C)**and hindlimb **(D)** from the 18.5 dpc stage embryos showing skeletal elements and maturation.

To investigate potential differences in endochondral ossification between control and *Elavl1* cKO embryos during development, skeletal preparations were performed at 15.5 and 18.5 dpc. No abnormalities were detected in the appendicular skeletal elements or their stage-specific ossification patterns in *Elavl1* cKO embryos compared to controls at either 15.5 dpc (Figure 3A) or 18.5 dpc (Figure 3B). Additionally, no differences were observed in the overall gross morphology at the corresponding developmental stages (Supplementary Figures 3A, B). All the skeletal elements in the stylopod, zeugopod, and autopod, both in forelimbs (Figure 3C) and hindlimbs (Figure 3D), from 18.5 dpc were analyzed. No aberrations in skeletal elements, including the scapula, humerus, radius, and ulna of the forelimb, femur, tibia, and fibula, were observed in Elavl1 conditional knockout limbs, clearly indicating the dispensability of Elavl1 during skeletal development.

**FIGURE 4:**
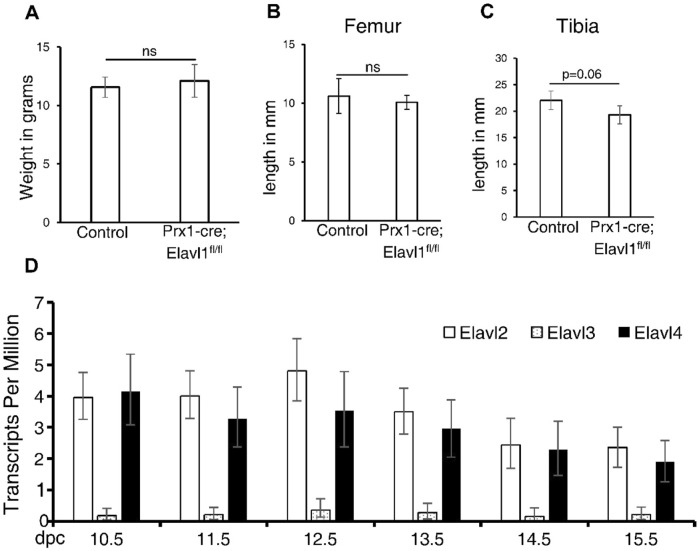
Body weight of 4-week-old male mice from control (N = 3, Elavl1^fl/fl^ and Prx1-cre; Elavl1^fl/+^) and Elavl1 cKO (N = 3) groups. p = 0.6; ns, not significant. **(A)** Femur **(B)** and tibia **(C)** lengths in the proximal–distal axis in 8-week-old male mice between control and Prx1-cre; Ealvl1^fl/fl^ mice; p = 0.59 (femur) and p = 0.06 (tibia). ns, not significant. **(D)** RNA-seq analysis of messenger RNA levels of Elavl2, Elavl3, and Elavl4 in limb RNA across developmental stages 10.5 dpc–15.5 dpc, indicating expression observed across these developmental stages.

In addition, we measured body weight and lengths of the tibia and femur at the indicated ages between control and Elavl1 cKO mice. There was no difference in the body weights between littermate controls and Elavl1 cKO animals (Figure 4A). In 8-week-old male and female mice, the lengths of femur (Figure 4B) and tibia (Figure 4C) from hindlimbs also did not differ significantly between controls and Elavl1 cKO mice.

Together, our results showed that Elavl1 is dispensable during appendicular skeletal development. Unlike other stem cell compartments, including in the bone marrow, intestine, and thymus, wherein Elavl1 plays crucial roles during tissue development and homeostasis, deletion in limb progenitor cells did not result in the disruption of skeletal patterning or morphological development. Deletion of Elavl1 globally during early development led to embryonic and extra-embryonic developmental defects with embryonic lethality post E14.5 dpc. Targeted embryonic deletion of Elavl1 in epiblast cells using Sox2-cre also revealed significant changes during embryonic development, affecting multiple tissues, including the spleen, skeletal tissues, and lungs, with no viable pups after birth (Kats et al., 2009). However, tissues such as the stomach and pancreas appeared normal in the absence of Elavl1 in conditional knockouts. Following tamoxifen-inducible Rosa26Cre-ERT2-mediated deletion of Elavl1 in 8-week-old mice, atrophy of multiple tissues was observed, even leading to death within 10 days after the administration of tamoxifen (Ghosh et al., 2009). These studies indicated differential tissue susceptibility to Elavl1 depletion during development. In this study, the *Elavl1* gene was deleted conditionally and in a tissue-specific manner in the developing limb buds using Prx1 enhancer-mediated cre expression for a more specific and localized deletion in developing limb buds and cranial mesoderm, where high expression of Elavl1 was detected. Viable Elavl1 conditional knockouts were obtained in expected Mendelian ratios during pre- and postnatal development, indicating the dispensability of Elavl1 during limb development. Furthermore, developmental and molecular analyses of embryos also did not reveal any morphological or structural changes in the limb during or after development. Analyzing the differentiation dynamics of Prx1-positive stem cells in Elavl1 conditional knockout mice could help identify subtle defects that may not manifest as overt phenotypic variations.

Elavl1 is a member of the Elavl protein family, which includes Elavl2, Elavl3, and Elavl4 (also known as HuR, HuB, HuC, and HuD, respectively). Unlike Elavl1, which is ubiquitously expressed, other Elavl family members show high expression in the nervous system, with little known about their developmental expression patterns and roles in other tissues (King et al., 1994; Good, 1995). Except Elavl3, all the other Elavl family members are expressed in developing limbs (Figure 4D). Elavl family members such as Elavl2 and Elavl4 are expressed in developing limbs during the same embryonic stages as Elavl1. Elavl2 knockout leads to incomplete lethality and growth retardation in mice postnatally (Kato et al., 2019), further showing neuronal independent functions for other Elavl1 family members. Further studies with compound mutations of Elavl family members will shed more light on the functions of specific members of this RNA-binding protein family during the development of various tissues.

## Data Availability

The original contributions presented in the study are included in the article/Supplementary Material; further inquiries can be directed to the corresponding author.

## Supplementary material

SUPPLEMENTARY FIGURE S1 Dot plots showing overlapping and distinct expression patterns of Elavl1 and Prrx1 in single-cell RNA (scRNA)-sequencing data (ENCSR062VQC) from embryonic forelimbs.

SUPPLEMENTARY FIGURE S2 Genotyping PCR showing a gel with wild-type and Elavl1 floxed alleles in the ear punch tissues from a litter.

SUPPLEMENTARY FIGURE S3 Representative whole-body images of 15.5 dpc **(A)** and 18.5 dpc embryos **(B)**. Scale bar, 5 mm.
