# Supplementary figures and images for: Elavl1 deletion in limb mesenchyme is dispensable for skeletal morphogenesis
Source: Front Cell Dev Biol. 2025 Jul 14;13:1501837. doi: 10.3389/fcell.2025.1501837 (PMC12301689; doi:10.3389/fcell.2025.1501837)

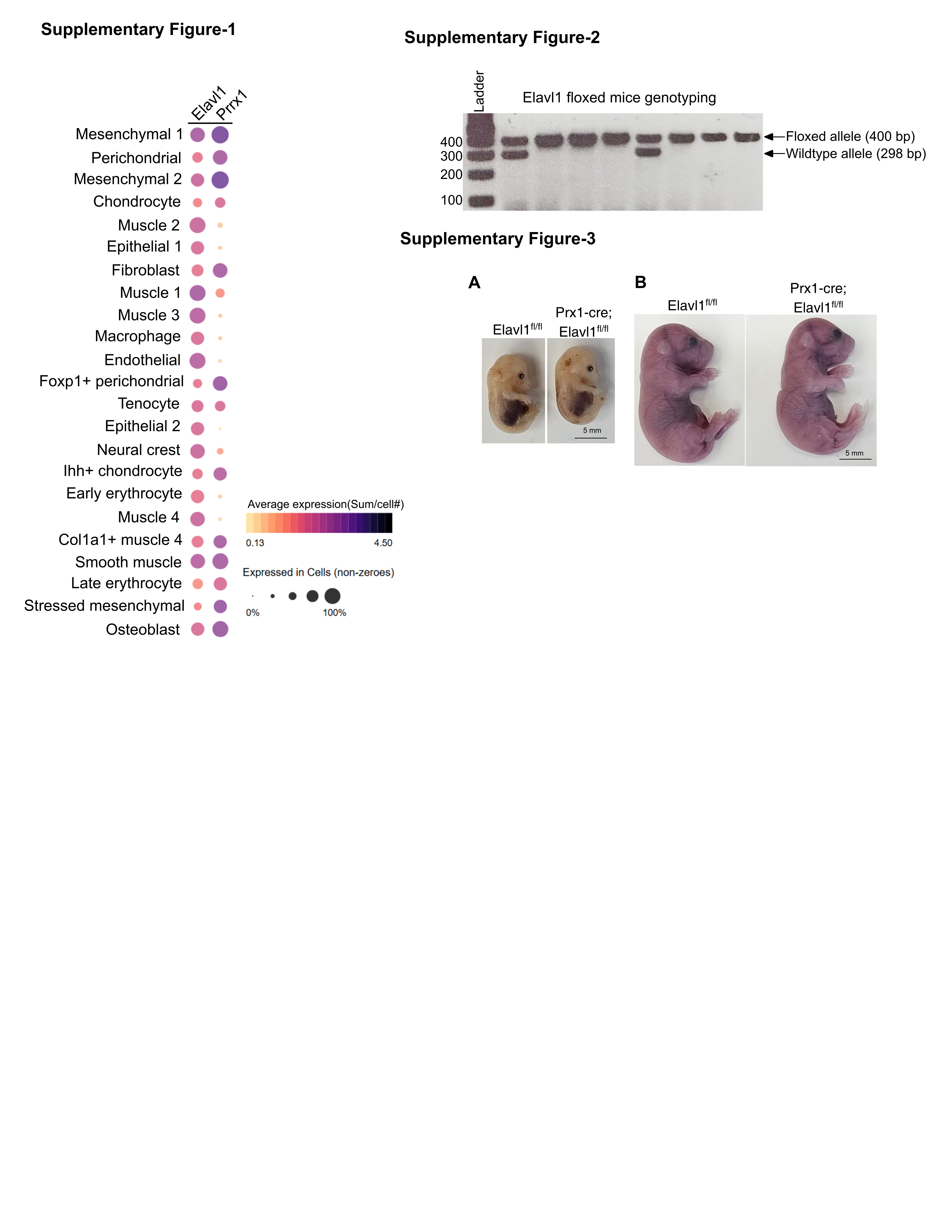

Supplement: Supplementary file 1 [file Image1.jpg]
